# Waterfowl—The Missing Link in Epidemic and Pandemic Cholera Dissemination?

**DOI:** 10.1371/journal.ppat.1000173

**Published:** 2008-10-31

**Authors:** Malka Halpern, Yigal Senderovich, Ido Izhaki

**Affiliations:** 1 Department of Biology Education, Faculty of Science and Science Education, University of Haifa, Oranim, Tivon, Israel; 2 Department of Evolutionary and Environmental Biology, Faculty of Science and Science Education, University of Haifa, Mount Carmel, Haifa, Israel; The Scripps Research Institute, United States of America

Cholera, a life-threatening diarrhoeal disease, has afflicted human beings and shaped human history for over two millennia. The disease still kills thousands of people annually. *Vibrio cholerae*, the etiologic agent of cholera, is endemic to aquatic environments [1], but despite intensive research efforts its ecology remains an enigma. The fatal effects of cholera are mainly due to the toxin produced by specific serogroups (O1 and O139) of *V. cholerae* [1]. Strains of *V. cholerae* that belong to serogroups other than O1 and O139, collectively referred to as the non-O1, non-O139 *V. cholerae*, have also been implicated as etiologic agents of moderate to severe human gastroenteritis [2]. The disease is endemic in Southern Asia and in parts of Africa and Latin America, where outbreaks occur widely and are closely associated with poverty and poor sanitation. The epidemic strains spread across countries and continents over time, giving rise to cholera pandemics [1]. It has been suggested that zooplankton function as a carrier of *V. cholerae* via ocean currents. However, the mechanism that enables *V. cholerae* to cross freshwater bodies within a continent, as well as oceans between continents, remains unknown. Here, we put forward a strongly neglected hypothesis that deserves more attention, and discuss evidence from the scientific literature that supports this notion: migratory water birds are possible disseminators of *V. cholerae* within and between continents.

**Figure 1:**
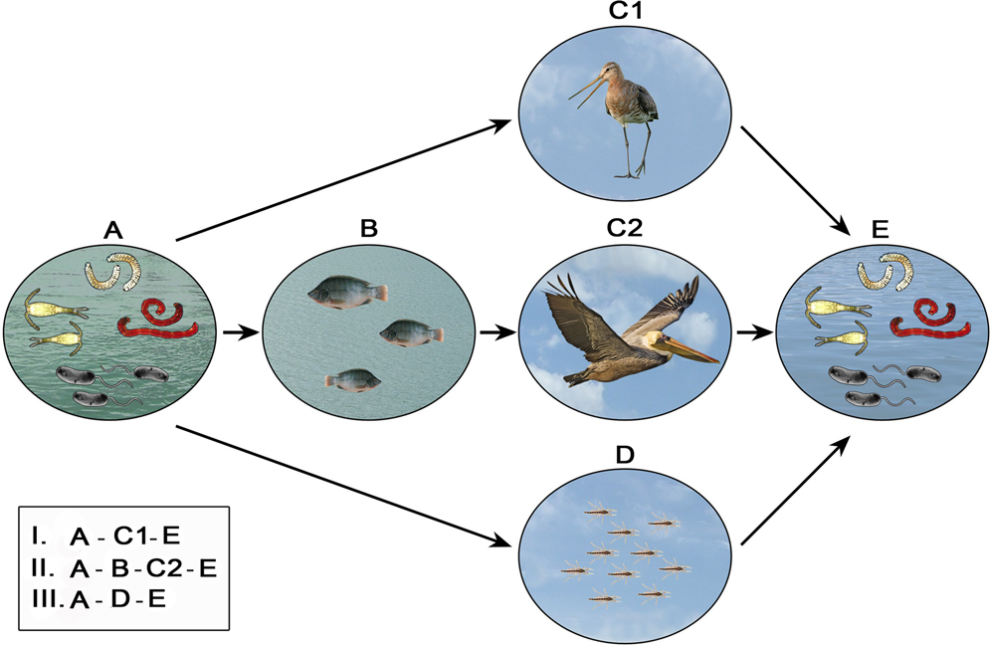
Three possible courses for the dissemination of *V. cholerae* between an endemic water body (A) and an uninfected water body (E). All three courses may exist in parallel. Course I: Copepods and chironomids, the main reservoirs of *V. cholerae* in fresh and marine ecosystems (A), may be consumed or carried by many species of waterfowl (e.g., waders) whose diet is based on insects and crustaceans (C1). These birds (C1) may serve as vectors for the dissemination of *V. cholerae* either by endozoochory (droppings) or by epizoochory (in the mud attached to their legs) into a new water body (E). Course II: Copepods and chironomids (A) may be consumed by various fish species (B) or by invertebrates such as mollusks, oysters, and crabs (not shown). Waterfowl such as pelicans and cormorants (C2) feed on the fish or the invertebrates or both, and hence may transfer the bacteria through their digestive tracts (endozoochory) into a new water body (E). Course III: Adult chironomids (D) directly carry *V. cholerae* between the two water bodies (A and E). This course has a limited range.

*V. cholerae* has been associated with crustaceans and especially copepods [1],[3],[4]. Copepod eggs hatch into nauplius larvae. The life cycle typically includes six naupliar stages and six copepodite stages, the last of which is the adult stage. These small crustaceans are found almost everywhere that water is available. Chironomids (Chironomidae, Diptera), also known as non-biting midges, are closely related to mosquitoes (Culicidae), but female chironomids do not bite or feed. They undergo complete metamorphosis of four life stages: eggs, larvae, pupae (aquatic stages), and adults that emerge into the air. Chironomids have also been found to serve as intermediate host reservoirs and possible windborne carriers for *V. cholerae* [5]–[7]. Although adult chironomids can fly and carry *V. cholerae* [8], they disperse over short distances of less than 1 km [9]. Dispersal of the adults by wind [8],[10] is restricted in its orientation and unlikely to be directed towards suitable habitats. Thus, chironomid movement by wind is probably not responsible for the long-distance dispersal of *V. cholerae* (Figure 1, course III).

Chironomids and copepods are abundant in aquatic ecosystems and are a major dietary component of many residential and migratory waterfowl [11]. Recently, reported evidence has suggested that larvae of *Chironomus salinarius* and Copepoda can survive the gut passage (endozoochory) in several bird species [12],[13]. The chironomid larvae were found to survive gut passage in the black-tailed Godwits (*Limosa limosa*) on autumn migration in southwest Spain [12]. Godwits and other waders move regularly over distances of up to 20 km between feeding and roosting sites while resting at stopover sites [14], thus facilitating passive dispersal between different water bodies within a wetland complex. Godwits fly at speeds of 60 km per hour [15], and could potentially disperse chironomid larvae over great distances during their migration between breeding areas in northern Europe and wintering areas in Africa [16] (Figure 1, course I).

Recent evidence indicates that viable copepods and chironomids are externally attached to birds' feet and feathers (epizoochory) [13]. Thus, endozoochorous and epizoochorous dispersal of these invertebrates via waterfowl may be a common phenomena and important process for *V. cholerae* dissemination (Figure 1, course I).

We recently isolated and identified *V. cholerae* non-O1 from the gut of several individual fish (*Tilapia* sp.) from various freshwater bodies in northern Israel (unpublished data). *Tilapia* is known to consume copepods and chironomids [17], and hence we assume that these food items, as well as other invertebrates, might well be the source of *V. cholerae* in the fish gut. Thus, we suggest that fish also function as intermediate reservoirs of *V. cholerae* (Figure 1, course II). Support for the finding that *V. cholerae* survive in fish comes from the fact that some cholera outbreaks have been correlated with the consumption of uncooked fish. Cholera was associated with the eating of salt fish, sardines, and other fish from an atoll lagoon [18]. Consumption of dried fish was significantly correlated with risk of cholera in Tanzania [19]. Three cases of cholera in Sydney, Australia, were reported in 2006. A food trace-back investigation revealed that the only factor common to all cases was the consumption of raw whitebait imported from Indonesia [20]. *V. cholerae* was isolated from fish called “lorna” (*Sciaena deliciosa*) that were caught in inshore waters in Peru during a Peruvian epidemic [21]. It was postulated that cholera endemicity in India was due to hilsa fish [22]. Moreover, seafoods, including mollusks, crustaceans, crabs, and oysters also feed on plankton and can become infected with *V. cholerae* [3],[4],[23]. Seafoods have been incriminated in cholera outbreaks in many countries, including the United States and Australia [24].

*Tilapia* species, from which *V. cholerae* has been isolated in Israel, are consumed by many waterfowl, such as pelicans, cormorants, herons, egrets, and gulls [25]. Furthermore, mollusks, crustaceans, crabs, and oysters are also consumed by waterfowl. Several of these waterfowl species are long-distance migratory birds. Pelicans, for example, cross three continents as they migrate in autumn from the Danube Delta in the western Black Sea region, pass through Israel to East Africa where they overwinter, and return to Europe in spring. During this journey they stop over at lakes and other water bodies in Turkey, Israel, Egypt, Sudan, Ethiopia, and Kenya [26]. We therefore suggest that waterfowl disperse *V. cholerae* not only as an outcome of their direct predation upon chironomids and copepods, but also because many of them consume fish and invertebrates. Thus, migratory waterbirds might carry *V. cholerae* between water bodies [4],[5] (Figure 1, course II).

Evidence that *V. cholerae* can survive in a bird's gut can be found in two studies that were published about twenty years ago but failed to attract the attention of the scientific community. In the first study [27], a survey was carried out between 1976 and 1979 in Kent, England, to establish the incidence of *V. cholerae* in the aquatic environment. *V. cholerae* was detected in 6% (15 out of 245) of cloacal swabs taken from gulls caught at times when *V. cholerae* could not be isolated from water. In the second study, *V. cholerae* was isolated from fecal specimens collected from 20 of 28 species of aquatic birds in Colorado and Utah during 1986 and 1987 [28]. Three serotype O1 biovar eltor subtype Ogawa isolates were recovered from *Ardea herodias* (great blue heron) and *Larus delawarensis* (rige-billed gull). Only non-O1 *V. cholerae* was detected in water samples collected from the birds' habitats. The authors could not explain the bird's source of infection with the epidemic *V. cholerae* O1 strains. Non-O1 serogoups were isolated from pelicans, herons, gulls, cormorants, and many other species [28]. The highest incidence of isolations of *V. cholerae* from bird feces occurred in spring and autumn [28]. Seasonality with high *V. cholerae* numbers in the spring and autumn was also found when *V. cholerae* numbers were monitored in chironomid egg masses [6]. Two distinct seasons were also described for copepod production, from February through April, and during the months of August and September [1]. All of this is in accordance with seasonal pattern of cholera outbreaks [1]. Few other studies have documented the presence of non-O1 *V. cholerae* in birds. *V. cholerae* has been detected in geese [29] and in gulls [30].

*V. cholerae* from different serogroups, including the pathogenic O1 serogroup, were isolated from waterbirds [27]–[30]. In some samples, non-O1 as well as O1 serogroups were isolated from the same specimen [28]. As far as we know, there is no evidence that *V. cholerae* cause any kind of disease in birds. *Vibrio cholerae* was isolated from samples of gulls that all appeared to be healthy [27]. Though still to be confirmed, it is a likely possibility that these bacteria are part of the normal flora of the bird gut. Also, the kind of relationship (commensalism, parasitism, or mutualism) between the bacteria and the birds has yet to be determined. Recently developed molecular tools for the detection of cholera toxin, pathogenic serogroups, and strain fingerprinting of *V. cholerae* (e.g., [31],[32]) should enable the detection of various *V. cholerae* strains in birds even without growing the bacteria in culture.

From the point of view of public health, wild birds are important because they carry emerging zoonotic pathogens, either as reservoir hosts or by dispersing infected arthropod vectors [33]. In addition, bird migration across national and intercontinental borders provides a mechanism for the establishment of new endemic foci of disease at great distances from the source of the infection. Waterfowl, for example, are asymptomatic carriers of influenza A virus, *Salmonella*, *Campylobacter jejuni*, and *Borrelia burgdorferi* (Lyme disease) [33].

Taken together, the above findings all support the hypothesis that migratory waterbirds are the disseminators of *V. cholerae* between water bodies, both within and between continents. Waterfowl may therefore be the missing link in understanding the cause of cholera dissemination all over the world. Knowledge of the species of waterfowl that carry *V. cholerae* and their migration patterns might thus be useful in helping to predict future outbreaks of cholera.
